# Supplementary material for: Content-rich biological network constructed by mining PubMed abstracts
Source: BMC Bioinformatics. 2004 Oct 8;5:147. doi: 10.1186/1471-2105-5-147 (PMC528731; doi:10.1186/1471-2105-5-147)
Supplement: Additional File 2 — The original results of the above study (non-essential files are deleted to keep the file size under the limit set by BMC bioinformatics). [file 1471-2105-5-147-S2.bz2 › chilibotAdditionalFile2/dip05/137ID7816143E492/html/FOS_JUN.html]

 


 **FOS** and **JUN** 
  
Found 4259 abstracts in PubMed, retrieved 05.  
 

 What does Google say? 
 PDF only 
| .edu only 

---

**Interactive relationship** (e.g. stimulation, inhibition, etc)

**Inhibitory relationship**- Gene expression for  **Fos**  was suppressed at both times, while that for Fosl1 and  **Jun**  was augmented at 12 h and suppressed at 48 h.  Ref: 12875578 Acta Otolaryngol, 2003
**Neutral relationship**

**Non-interactive relationship** (e.g. studied together, co-existance, homology, etc.)

- MATERIAL AND METHODS From cDNA microarray screening of those specimens, several gene clusters, including Nos2 and the transcription factors  **Fos** , Fosl1,  **Jun**  and Nfkb1, were identified as possible upstream regulators of Na transport protein expression.  Ref: 12875578 Acta Otolaryngol, 2003
- In these mice context dependent memory retrieval evoked a marked induction of c  **Fos**  and JunB, but not of c  **Jun**  and JunD, in pyramidal CA1 neurons of the dorsal hippocampus.  Ref: 12882314 Genes Brain Behav, 2003
- CHOP dimerizes with and inhibits the binding of C EBP related transcription factors to their consensus DNA target sequences and also forms novel complexes with other transcriptional proteins e.g. c  **jun** , c  **fos** .  Ref: 12876286 J Biol ChemJ Biol Chem, 2003
- These results demonstrate that contextual memory retrieval is associated with de novo synthesis of specific members of the  **Fos**   **Jun**  transcription factor family.  Ref: 12882314 Genes Brain Behav, 2003
- Using specific polyclonal antisera against c  **Fos** , JunB, c  **Jun**  and JunD, we tried to identify the candidate transcription factors of the immediate early gene family which may contribute to the molecular processes during contextual memory reconsolidation.  Ref: 12882314 Genes Brain Behav, 2003
- Both VPA and lithium altered the expression of the early inducible genes for c  **fos**  and c  **jun**  thus promoting the expression of specific proteins.  Ref: 12890311 Int J Neuropsychopharmacol, 2003
- The inhibition of S phase induction was associated with down regulation of c Myc, but not of c  **Fos**  or c  **Jun** .  Ref: 12883011 Proc Natl Acad Sci U S AProc Natl Acad Sci U S A, 2003
